# Association of Pancreatic Polypeptide with Mild Cognitive Impairment Varies by APOE ε4 Allele

**DOI:** 10.3389/fnagi.2015.00172

**Published:** 2015-09-08

**Authors:** Rosebud O. Roberts, Jeremiah A. Aakre, Ruth H. Cha, Walter K. Kremers, Michelle M. Mielke, Stefanie N. Velgos, Yonas E. Geda, David S. Knopman, Ronald C. Petersen

**Affiliations:** ^1^Division of Epidemiology, Department of Health Sciences Research, Mayo Clinic, Rochester, MN, USA; ^2^Department of Neurology, Mayo Clinic, Rochester, MN, USA; ^3^Division of Biomedical Statistics and Informatics, Department of Health Sciences Research, Mayo Clinic, Rochester, MN, USA; ^4^Center for Clinical and Translational Science, Mayo Clinic, Rochester, MN, USA; ^5^Department of Psychiatry and Psychology, Mayo Clinic, Scottsdale, AZ, USA; ^6^Department of Neurology, Mayo Clinic, Scottsdale, AZ, USA

**Keywords:** cognition, mild cognitive impairment, case–control study, pancreatic polypeptide, neuropeptide, type 2 diabetes, apolipoprotein E

## Abstract

We conducted a preliminary case–control investigation of the association of pancreatic polypeptide (PP) with mild cognitive impairment (MCI) in 202 MCI cases (mean age, 81.6 years) and 202 age- and sex-matched cognitively normal controls in the Mayo Clinic Study of Aging. Plasma PP was measured and examined as the natural logarithm (continuous) and dichotomized at the median. The OR (95% CI) of MCI increased with increasing PP [1.46 (1.04–2.05)]. There was a negative interaction of PP with apolipoprotein E (APOE) ε4 allele; compared to the reference group (no APOE ε4 allele and low PP), the OR (95% CI) for combinations of ε4 and PP were: 2.64 (1.39–5.04) for APOE ε4 plus low PP; 2.09 (1.27–3.45) for no APOE ε4 plus high PP; and 1.91 (1.04–3.53) for no APOE ε4 plus high PP (*P* for interaction = 0.017). There was also a trend toward a negative interaction with type 2 diabetes (*P* for interaction = 0.058). Compared to no diabetes and low PP, the OR (95% CI) was 3.02 (1.22–7.46) for low PP plus diabetes but 1.80 (1.01–3.22) for high PP plus diabetes. Participants with high PP had a greater mean (SD) weight loss (kilograms per decade) than persons with low PP [−2.27 (4.07) vs. −1.61 (5.24); *P* = 0.016]. MCI cases had a non-significantly greater weight loss per decade compared to controls. These findings suggest that high PP alone or jointly with APOE ε4 allele or type 2 diabetes is associated with MCI, and that high PP may mitigate some effects of APOE ε4 allele and type 2 diabetes on cognition. Potential mechanisms may involve PP-related weight loss and centrally mediated effects of PP on cognition. These findings remain to be validated in other studies.

## Introduction

The need for diagnostic and prognostic biomarkers remains essential for early detection and prevention of Alzheimer’s disease (AD). Blood-based (non-genetic) biomarkers are important because they are easily acquired, relatively inexpensive compared to brain imaging biomarkers, less invasive than cerebrospinal fluid (CSF) acquisition, and more amenable to large-scale screening. Blood-based biomarkers may have utility for early detection or enhance recruitment and monitoring in clinical trials (Doecke et al., 2012).

Pancreatic polypeptide (PP) has been associated with mild cognitive impairment (MCI) and AD dementia in blood-based biomarker panels for these conditions (Craig-Schapiro et al., 2011; O’Bryant et al., 2011; Doecke et al., 2012). PP is produced by F cells of the pancreatic islets of Langerhans and released into the circulation after food ingestion. It is one of several neuropeptides with activity both in the gut and in the brain regions affected by AD dementia, such as the hippocampus, and hypothalamus (Asakawa et al., 2003). The effects of secreted PP are mediated by neuropeptide receptors (Y4 and Y5) in the brain and in the gut through vagal signaling. In response to PP, there is vagal signaling to neuroendocrine regions of the brain, such as the hypothalamus, locus ceruleus, area postrema, dorsal vagal complex, and brain stem regions that control gastrointestinal functions and regulate food intake (Holzer et al., 2012; Asakawa et al., 2003; McTigue et al., 1993). Physiologic effects of PP in response to vagal signaling from the brain include decreased gastric emptying, appetite suppression, decreased food intake, and a negative energy balance (i.e., energy expenditure exceeds energy intake) (Asakawa et al., 2003; Holzer et al., 2012). Low physiologic levels of PP and low food-induced PP are associated with hyperphagia and morbid obesity (Lassmann et al., 1980; Marco et al., 1980); in contrast, administration of PP stimulates weight loss (Berntson et al., 1993). Because of the association of high levels of PP with MCI and AD dementia in biomarker panels using multiplex platforms, the association of high levels of PP with reduced food intake and weight loss, and the importance of diet and caloric intake in risk of MCI and AD, we hypothesized that abnormally increased PP levels may be involved in the pathogenesis of MCI. The objective of this study was to conduct a preliminary cross-sectional investigation of plasma PP with MCI among participants in the Mayo Clinic study of aging (MCSA).

## Materials and Methods

### Study design and participants

The details of the study design and methodology for participant recruitment have been published (Roberts et al., 2008). Briefly, the MCSA is a population-based study established in Olmsted County, MN, USA, in 2004. Participants aged 70–89 years were randomly selected from an enumeration of the county population through the Rochester Epidemiology Project (Rocca et al., 2012), a medical records linkage system. We excluded subjects who were terminally ill or in hospice, or had previously diagnosed dementia. Participants were clinically evaluated to assess cognitive status (normal cognition, MCI, or dementia). Ongoing recruitment was established in 2008 to maintain the sample size, and follow-up is performed every 15 months. All study protocols were approved by the institutional review boards of the Mayo Clinic and Olmsted Medical Center. All participants provided written informed consent prior to participation.

The clinical evaluation included an interview of the participant (using questions about memory) and an informant (using the Functional Activities Questionnaire and Clinical Dementia Rating Scale) (Pfeffer et al., 1982; Morris, 1993) by a study coordinator to evaluate memory and functioning; a complete neurologic evaluation by a physician, which included the Short Test of Mental Status – a global test of memory (Kokmen et al., 1987) and a full neurological evaluation; and a neuropsychological testing battery consisting of nine tests to assess performance in four cognitive domains: memory (three tests), executive function (two tests), visuospatial skills (two tests), and language (two tests) (Roberts et al., 2008; Petersen et al., 2010). Data from the evaluation were reviewed for a diagnosis of MCI defined as: (i) cognitive concern; (ii) impairment in one or more cognitive domains; (iii) essentially normal functional activities; and (iv) absence of dementia (Petersen, 2004; Roberts et al., 2008; Petersen et al., 2009, 2010), taking into account level of education and longest held occupation. A diagnosis of dementia was based on DSM IV criteria (American Psychiatric Association, 2000; Petersen, 2004; Roberts et al., 2008; Petersen et al., 2009, 2010); diagnosis of normal cognition was made in persons who performed in the normal cognitive range and did not meet criteria for MCI or dementia (Roberts et al., 2008; Petersen et al., 2010).

### MCI cases and controls

Cases and controls for the present study were selected from among participants who were evaluated prior to December 31, 2011, and had stored blood samples available for measurement of PP. Among 1,323 eligible participants, 202 had MCI. Each case was matched to a cognitively normal control by sex, age (±2 years), number of clinical visits in the MCSA (±1), and duration of follow-up (±1 year; 1:1 matching).

### Measurement of PP

Pancreatic polypeptide was measured using a radioimmunoassay technique developed in the Mayo Clinic Endocrine Laboratory (Schwartz, 1983; Koch et al., 1985) The assay included a commercial antibody from Peninsula Laboratories International, Inc., radioactive reagents from PerkinElmer Inc., and calibration material from Sigma-Aldrich. The assay system utilized rabbit antihuman PP antiserum, a standard or patient plasma specimen, and radiolabeled human PP, which has been iodinated by a modified Hunter–Greenwood technique. The sample and antibody (both primary and secondary) and the radioactive label were diluted in the same buffer. The buffer consisted of 0.02 M sodium 5,5-diethylbarbiturate (Sigma B-050), 4.12 g, 0.006 M barbituric acid (Fisher CAS#67527), 0.02% bovine serum albumin and some preservatives dissolved in water, and the pH was adjusted to 8.2. The buffer and precipitating antibody and validation were performed in the laboratory. The standard (patient) HPP competes with the radioactive HPP for binding sites on the primary antibody, which is rabbit anti-human pancreatic polypeptide. This antibody complex was precipitated with Goat Anti Rabbit serum along with normal rabbit serum and polyethylene glycol. The mixture was centrifuged and the supernate discarded. The radioactive counts in the pellet are inversely proportional to the amount of HPP present in the tube. The 2.5 SD detection limit based on 20 determinations from one assay was 28.5 pg/mL. The coefficients of variation were 3.65% for intra-assay precision and 8.05% for interassay precision.

### Other covariates

Demographic variables were assessed at baseline by interview. Medical comorbidities (type 2 diabetes mellitus, hypertension, stroke, and coronary artery disease) and maximum adult weight (age 40–65 years) were ascertained from medical records of each participant using the Rochester Epidemiology Project. Apolipoprotein E (APOE) genotyping was performed from a blood draw using standard methods (Hixson and Vernier, 1990), and depressive symptoms were ascertained from the Neuropsychiatric Inventory Questionnaire (Kaufer et al., 2000).

### Statistical analyses

The distribution of PP in cases and controls was skewed, so we characterized PP using the natural logarithm. We also examined PP as a dichotomous variable, with a high PP (considered abnormal) arbitrarily defined using a median split and the 90th percentile based on the distribution of PP levels for cognitively normal controls. We examined the associations of PP with MCI using conditional logistic regression models matched on sex and age (basic model) and also adjusted for age at sample draw to account for any residual confounding by age. In separate models, we investigated potential confounding of the association of PP with MCI by covariates that were significantly associated with MCI [education, depression, hypertension, coronary artery disease, type 2 diabetes, and APOE ε4 genotype (any ε4 vs. no ε4 allele)]; there was no association with body mass index (BMI). We examined effect modification by including interaction terms of each covariate with PP (dichotomized as ≤median vs. >median) in models with the main effects. To determine whether PP was associated with weight loss prior to the date of blood draw, we computed the average weight loss (in kilograms) per decade from the maximum weight in midlife to date of blood draw for PP assessment in MCI cases vs. controls and for participants with high vs. low PP levels. Comparisons across groups were made using Wilcoxon rank sum tests. All *P* values were considered significant at alpha of 0.05. Analyses were performed by SAS version 9.3 (SAS Institute).

## Results

The characteristics of MCI cases and controls are described in Table 1. The mean age was 81.6 years and 58.9% were men. Cases had significantly fewer years of education; higher frequency of type 2 diabetes, hypertension, coronary artery disease, depressive symptoms, and APOE ε4 allele status; and marginally higher PP levels compared to controls (*P* = 0.073).

**Table 1:** **Characteristics of study participants**.

Characteristic	Normal cognition (*N* = 202)	MCI (*N* = 202)
Male sex, no. (%)	119 (58.9)	119 (58.9)
Age, mean (SD) (years)	81.6 (5.8)	81.6 (5.8)
Education, mean (SD) (years)^a^	14.4 (2.8)	13.7 (3.0)
APOE ε4 allele, no. (%)^b^	54 (26.7)	70 (34.7)
BMI, mean (SD)	27.2 (4.3)	27.2 (4.8)
Diabetes, no. (%)^a^	39 (19.3)	59 (29.2)
Hypertension, no. (%)^a^	158 (78.2)	174 (86.1)
Coronary artery disease, no. (%)^a^	66 (32.7)	88 (43.6)
Depressive symptoms, no. (%)^a^	11 (5.4)	23 (11.4)
**PP**
Median PP (IQR)^b^	196 (141.0,308.0)	229 (148.0,343.0)
Ln PP median (IQR)^b^	5.28 (4.95,5.73)	5.43 (5.00,5.84)
>50th percentile (median), no. (%)^b^	100 (49.8)	119 (59.2)
>90th percentile, no. (%)^b^	20 (10.0)	32 (15.9)

*BMI, body mass index; IQR, interquartile range; ln, natural logarithm; MCI, mild cognitive impairment; PP, pancreatic polypeptide*.

*Percentages are based on non-missing numbers*.

*^a^*P* ≤ 0.05*.

*^b^0.05 < *P* < 0.10*.

*Pancreatic polypeptide level was missing in one control and one case*.

### Cross-sectional association of PP with MCI

In age- and sex-adjusted models, the OR of MCI increased with increasing PP level [β (SE), 0.379 (0.173); *P* = 0.029] (Table 2). When participants were characterized by dichotomous cut points, the associations of high PP with MCI persisted but were marginally significant: OR, 1.47 (*P* = 0.069) for PP greater than median and OR, 1.89 (*P* = 0.051) for PP >90th percentile (Table 2). There was no confounding by coronary artery disease, type 2 diabetes, hypertension, depressive symptoms, or APOE ε4 allele when each variable was separately included in a model with age and sex (data not shown). When all the potential confounders were included in the model, the OR for MCI remained elevated but decreased to 1.33 (*P* = 0.116).

**Table 2:** **Conditional logistic regression models for association of pancreatic polypeptide with mild cognitive impairment**.

	Model 1^a^		
	***β*** (SE)	OR (95% CI)	*P* value
Ln PP	0.379 (0.173)	1.46 (1.04–2.05)	0.029
PP > median^b^	0.388 (0.214)	1.47 (0.97–2.24)	0.069
PP > 90th percentile^c^	0.637 (0.327)	1.89 (1.00–3.59)	0.051

*ln, natural logarithm; PP, pancreatic polypeptide*.

*^a^Model 1: Conditional logistic regression models adjusted for education. Participants were matched on age and sex*.

*^b^Median PP, 196 pg/mL*.

*^c^90th percentile, 452 pg/mL*.

**Figure 1:**
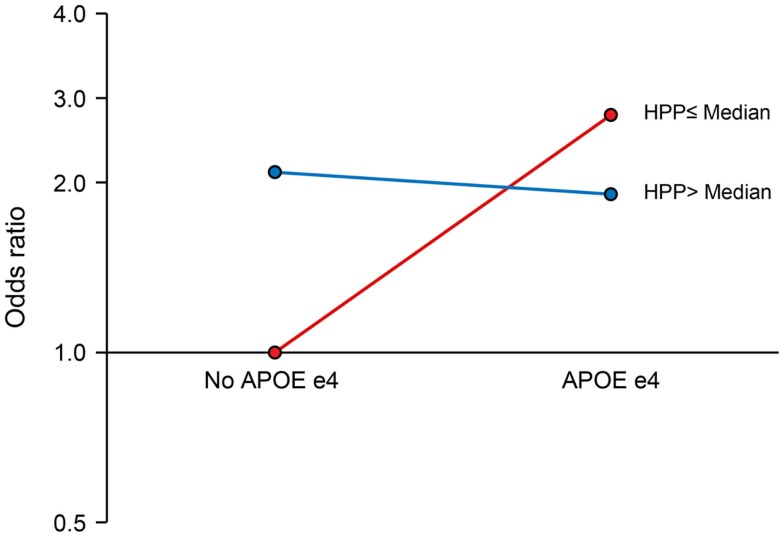
**Interaction of pancreatic polypeptide (PP) with APOE ***ε***4 Allele**. Compared to persons without an APOE ε4 allele and low PP level (reference), the ORs (95% CIs) were as follows: 2.64 (1.39–5.04), *P* = 0.003 for low PP plus APOE ε4 allele; 2.09 (1.27–3.45), *P* = 0.004 for high PP without APOE ε4 allele; and 1.91 (1.04–3.53), *P* = 0.038 for high PP plus APOE ε4 allele. PP was dichotomized at the median as low PP (≤196 pg/mL) and high PP (>196 pg/mL).

**Figure 2:**
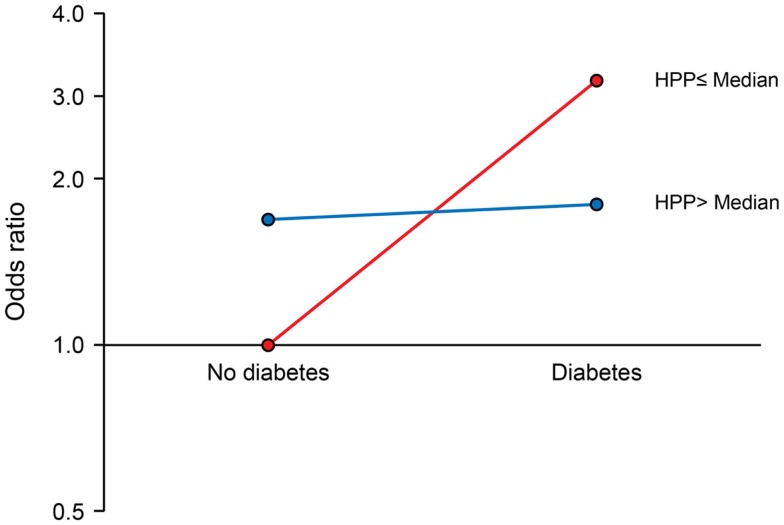
**Interaction of pancreatic polypeptide (PP) with type 2 diabetes**. Compared to persons without type 2 diabetes and low PP level (reference), the ORs (95% CIs) were as follows: 3.02 (1.22–7.46), *P* = 0.017 for low PP plus diabetes; 1.69 (1.07–2.68), *P* = 0.026 for high PP without diabetes; and 1.80 (1.01–3.22), *P* = 0.046 for high PP plus diabetes. PP was dichotomized at the median as low PP (≤196 pg/mL) and high PP (>196 pg/mL).

We observed a significant interaction of PP with APOE ε4 allele (*P* for interaction = 0.017). The joint effects of high PP and APOE ε4 allele were indicative of a negative (antagonistic) interaction. Compared to the reference group (no APOE ε4 allele and low PP), the ORs (95% CIs) for combinations of APOE and PP were as follows: 2.64 (1.39–5.04), *P* = 0.003 for APOE ε4 plus low PP; 2.09 (1.27–3.45), *P* = 0.004 for no APOE ε4 plus high PP; and 1.91 (1.04–3.53), *P* = 0.038 for APOE ε4 plus high PP (Figure 1). There was also a marginal negative interaction with type 2 diabetes (*P* for interaction = 0.058). Compared to the reference group (no type 2 diabetes and low PP), the ORs (95% CIs) of MCI were: 3.02 (1.22–7.46), *P* = 0.017 for diabetes plus low PP; 1.69 (1.07–2.68), *P* = 0.026 for no diabetes plus high PP; and 1.80 (1.01–3.22), *P* = 0.046 for diabetes plus high PP (Figure 2).

### Weight loss

Mild cognitive impairment cases had a non-significantly greater mean (SD) weight loss (kilograms per decade) assessed from midlife to date of blood draw for measurement of PP compared to controls [−2.40 (5.14) vs. −1.53 (4.03); *P* = 0.124]. However, participants with high PP had a greater mean weight loss per decade than persons with low PP [−2.27 (4.07) vs. −1.61 (5.24); *P* = 0.016] and a non-significantly lower BMI [26.906 (4.73) vs. 27.534 (4.37); *P* = 0.212].

## Discussion

In this preliminary case–control study of elderly persons, elevated PP levels were associated with an increased OR of MCI. However, the association of PP with MCI varied significantly by APOE ε4 allele, and non-significantly, by diabetes status. High PP was also associated with greater weight loss per decade, and MCI cases had a non-significantly greater weight loss per decade compared to controls. The findings suggest that PP may be involved in the pathogenesis of MCI and should be examined in a definitive prospective study.

The negative interaction of PP with APOE ε4 allele and marginally significant interaction with diabetes are interesting, but the implications are unclear. The interactions showed that the OR for the joint effects of an APOE ε4 allele or diabetes with high PP was lower than that for APOE ε4 or diabetes with low PP (i.e., APOE ε4 or diabetes alone), but comparable to the elevated estimates of OR in APOE ε4 allele non-carriers and in non-diabetics.

Our findings are in keeping with findings from other investigators (Doecke et al., 2012; Hu et al., 2012; Soares et al., 2012; O’Bryant et al., 2013; Burnham et al., 2014). In the Australian Imaging Biomarker and Lifestyle study, PP levels were elevated 1.54-fold in AD dementia cases compared to cognitively normal controls (Doecke et al., 2012). In AD Neuroimaging Initiative samples/cohorts plasma, PP levels were higher in AD dementia or MCI cases compared to controls (Kiddle et al., 2012; Soares et al., 2012). In the Texas Alzheimer’s Research and Care Consortium, PP was overexpressed in a biomarker panel and improved the diagnostic accuracy for AD in whites (O’Bryant et al., 2011) and in Mexican Americans (O’Bryant et al., 2013). In two independent samples from the University of Pennsylvania and Washington University, elevated PP levels were associated with impaired cognition (MCI, AD dementia, or Clinical Dementia Rating of 0.5 or 1) (Hu et al., 2012).

Mechanisms that mitigate effects of elevated PP levels on MCI risk in APOE ε4 carriers may involve the need to maintain a balance in inhibitory and excitatory input in the hippocampus for memory encoding and spatial recognition (Andrews-Zwilling et al., 2010). This balance is maintained by somatostatin-expressing cells, which colocate with γ-aminobutyric acid-ergic (GABAergic) expression to provide inhibitory input on pyramidal cell activity in the hippocampus (Freund and Buzsaki, 1996). With aging, there is a decrease in somatostatin and GABAergic interneuron expression in the hippocampus; this results in increased excitatory activity at rest that is hypothesized to contribute to memory impairment in amnestic MCI (Yassa et al., 2010, 2011). This decreased somatostatin expression is also observed in AD brains and in APOE ε4 allele carriers (Kumar, 2005). Functional imaging studies have reported greater default brain network activation at rest and during memory tasks and greater hippocampal activation in persons at increased risk of genetic or familial AD, APOE ε4 allele carriers (Bookheimer et al., 2000; Bassett et al., 2006; Filippini et al., 2009), and in individuals with MCI (Dickerson et al., 2004, 2005; Celone et al., 2006; Hamalainen et al., 2007). These findings suggest that there is increased imbalance in inhibitory–excitatory signals and impaired inhibition of GABAergic interneurons in persons at risk for AD, such as persons with MCI and ε4 carriers. This APOE ε4 effect may be exacerbated in elderly ε4 carriers who also have age-related decrease in somatostatin, resulting in greater excitatory activity in granule and pyramidal cells in the hippocampal formation and impaired memory encoding compared to non-carriers (Andrews-Zwilling et al., 2010). In aMCI patients, administration of antiepileptic drugs reduced CA3/dentate overactivity and improved memory performance (Bakker et al., 2012).

Similarly, animal studies have demonstrated decreased somatostatin in mouse models of age-related neurodegeneration (Andrews-Zwilling et al., 2010) and in the CA3/dentate gyrus of aged rats with age-related loss of spatial memory involving the hippocampus (Spiegel et al., 2013). A recent study in mice demonstrated that PP activates somatostatin-expressing cells in the hippocampus (Kim et al., 2014). Other animal studies have also reported beneficial effects of antiepileptic drugs and inhibitory neuropeptides that target loss of GABAergic activity on memory performance in memory-impaired rats compared to controls (Koh et al., 2010, 2013). APOE ε4 impairs GABAergic interneurons, increases excitatory activity in the hippocampus, and impairs memory encoding (Andrews-Zwilling et al., 2010). Pentobarbital was observed to improve performance in APOE ε4 knock-in mice with deficits in hippocampal inhibitory activity (Andrews-Zwilling et al., 2010). Together, these human and animals studies are highly relevant to our findings and are in keeping with the interaction of PP with APOE ε4 allele observed in our study. If indeed PP (a neuropeptide) activates somatostatin-containing cells in persons at risk for AD (e.g., APOE ε4 carriers), elevated levels of PP in APOE ε4 carriers may reduce excitatory signals, reduce the inhibitory–excitatory imbalance to GABAergic neurons, and promote memory encoding.

In persons with diabetes, elevated PP levels may reduce the likelihood of MCI by effects on weight reduction, negative energy balance, and improved glycemic control. Effects of PP are mediated by binding to neuropeptide Y (NPY) receptors in the gastrointestinal tract, pancreas, liver, and brain (hippocampus and hypothalamus). High PP levels may increase insulin secretion from the pancreatic islets of Langerhans cells through inhibition of somatostatin release (Mandarino et al., 1981; D’Alessio et al., 1989; Kim et al., 2014). Elevated levels of PP secretion observed in diabetics (Floyd et al., 1976) is hypothesized to be a compensatory mechanism to increase insulin release and improve glycemic control (Floyd et al., 1976; Kim et al., 2014); this may also enhance brain insulin signaling and glucose metabolism, and thereby reduce MCI risk (Roberts et al., 2013). Central effects of PP involve the hypothalamus, dorsal vagal complex (McTigue et al., 1993). High circulating PP may enhance the brain control of gastrointestinal functions (McTigue et al., 1993; Asakawa et al., 2003). PP inhibits hypothalamic release of neuropeptides that stimulate eating, enhances release of anorexigenic hypothalamic peptides, and suppresses gastric release of ghrelin (an appetite stimulant) (Asakawa et al., 2003), with potentially beneficial effects in diabetics.

In APOE ε4 non-carriers and non-diabetics, the potential mechanisms for the association of high PP levels with MCI are unclear, but may relate to dietary effects of PP. Overexpression of PP may lead to decreased food intake, excessive weight loss, anemia, and deficiencies in micronutrients required for neuronal function. Consistent with this, we observed a non-significantly greater weight loss per decade in MCI cases compared to controls, in keeping with reported declines in weight prior to dementia (Nourhashemi et al., 2003; Knopman et al., 2007; Besser et al., 2014; Sobow et al., 2014). Persons with high PP levels in the study also had a greater average weight loss and lower BMI than persons with lower PP levels. Nutritional deficiencies have adverse implications for cognition. Several dietary nutrients and antioxidants including vitamins (e.g., A, C, D, E, folate, B12), monounsaturated and polyunsaturated fatty acids, dietary antioxidants, and phospholipids (Blok et al., 1996; Engelhart et al., 2002; Chrysohoou et al., 2004; Feart et al., 2009; Roberts et al., 2010a,b) are beneficial for cognitive function. Deficiencies in micronutrients reportedly occur prior to the protein-calorie malnutrition observed in patients with AD dementia (Lopes da Silva et al., 2014). Dietary nutrients are the basis for ongoing AD dementia prevention and treatment trials (Scheltens et al., 2010; Hartmann et al., 2014; Swaminathan and Jicha, 2014). Elevated PP levels have been observed in persons with both AD and non-AD dementia (Hu et al., 2012). The association with non-AD dementia, which may have a vascular etiology, suggests that vascular mechanisms, possibly involving diet-related metabolic or signaling abnormalities, may be involved in the association of PP with cognitive impairment. There are also suggestions that elevated PP levels may be part of an immune signature that may adversely affect MCI risk (Hallgren and Lundqvist, 1980; Ray et al., 2007; Burnham et al., 2014).

The cause of the abnormally elevated levels of PP is uncertain. There are suggestions that this could result from a dysfunction in cholinergic tone that precedes and is present in clinical AD dementia (Mufson et al., 2008; Doecke et al., 2012; Hu et al., 2012; Soares et al., 2012; O’Bryant et al., 2013; Burnham et al., 2014). Elevated levels of PP observed in CSF of patients with AD suggest that impaired transport across the blood–brain and CSF barriers may occur (Hu et al., 2010, 2012). A disruption in the blood–brain barrier in the aging hippocampus could also lead to elevated brain PP levels (Hu et al., 2012; Montagne et al., 2015).

A limitation of our study is that the case–control design precludes our ability to assess causality. However, these preliminary findings have generated hypotheses for further investigations. Another limitation is that several gut hormones that influence energy balance may interact with PP; however, as an initial step, we specifically examined only PP. Another limitation is that due to the imbalance in MCI risk factors between cases and controls, the ORs were attenuated in the multivariable models and the associations were no longer significant. Finally, several complex metabolic and neuronal pathways and mechanisms are involved in the association of PP with cognition and we are limited in our ability to fully investigate these associations using a case–control design.

Our study has several strengths. Participants were well characterized for cognitive outcomes. We adjusted for the effects of vascular risk factors using information from the medical record. In contrast to several studies, we measured PP levels from overnight fasting blood samples to eliminate potential confounding by food intake. Given the prospective design of the MCSA, we are able to conduct a definitive study to test the hypotheses generated by this preliminary investigation.

## Author Contributions

RR designed the study; RR, MM, DK, RP, WK, JA, RC acquired, analyzed, and interpreted the data; RR drafted the manuscript; RR, MM, DK, RP, WK, JA, YG, SV critically reviewed the manuscript for intellectual content; WK, RC, JA performed the statistical analyses; RP, RR, MM, DK obtained the funding; RP and RR provided administrative, technical, or material support; RR and WK provided study supervision.

## Conflict of Interest Statement

The authors declare that the research was conducted in the absence of any commercial or financial relationships that could be construed as a potential conflict of interest.

## Funding

The study was supported by the National Institute on Aging (U01 AG006786, P50 AG016574), the Mayo Foundation for Medical Education and Research, and was made possible by the Rochester Epidemiology Project (R01 AG034676).

## Abbreviations

AD, Alzheimer’s disease; APOE, apolipoprotein E; BMI, body mass index; CF, cerebrospinal fluid; MCI, mild cognitive impairment; MCSA, Mayo Clinic Study of Aging; PP, pancreatic polypeptide.
